# Developing a lifetime disease model from RCT data using parametric models with time-updated covariates

**DOI:** 10.1186/1745-6215-14-S1-O24

**Published:** 2013-11-29

**Authors:** Iryna Schlackow, Borislava Mihaylova

**Affiliations:** 1 University of Oxford, Oxford, UK

## Aims

Patients with moderate to advanced chronic kidney disease (CKD) are at increased risk of cardiovascular (CV) events, which, in turn, accelerate CKD progression. We use data from the 9,270-patient Study of Heart and Renal Protection (SHARP) to develop a disease model that takes into account the interdependence between CKD progression and CV risk over time and can be used to simulate disease outcomes and life expectancy over lifetime.

## Methods

Participants’ characteristics at baseline (sociodemographic and CV and CKD risk factors) as well as their progression through CKD stages and experience of CV events during the study were related to the CV and CKD disease endpoints using parametric survival regressions (CV endpoints) or multinomial and logistic regressions (CKD progression). The risk equations were combined into an interacted Markov decision analytical model.

## Results

In all CKD and CV risk equations, within-trial non-fatal CV events and contemporary CKD stages were major drivers of subsequent CKD and CV outcomes. The time-updated non-fatal outcomes captured well the increased hazard of death but further temporal parameterization of baseline hazard was indicated in modeling the combined non-fatal and fatal CV endpoints. The decision model predictions were concordant with disease event rates observed among all study patients and in important subgroups.

## Conclusions

Major non-fatal disease events are important drivers of health outcomes and costs. Inclusion of these events as time-updated covariates is likely to reduce reliance on ancillary parameters and produce more robust lifetime predictions. Long-term data are needed to discriminate between closely fitting risk models.

